# Long-term functional outcomes of patients with Hirschsprung disease following pull-through

**DOI:** 10.1186/s12887-022-03301-6

**Published:** 2022-05-03

**Authors:** Gunadi, Theodora Monica Carissa, Stevie, Ezzah Fatmala Daulay, Dicky Yulianda, Kristy Iskandar, Andi Dwihantoro

**Affiliations:** 1 Pediatric Surgery Division, Department of Surgery, Faculty of Medicine, Public Health and Nursing, Universitas Gadjah Mada/Dr. Sardjito Hospital, Jl. Kesehatan No. 1, Yogyakarta, 55281 Indonesia; 2 Department of Child Health, Faculty of Medicine, Public Health and Nursing, Universitas Gadjah Mada/UGM Academic Hospital, Yogyakarta, 55291 Indonesia

**Keywords:** Bowel function score, Definitive surgery, Hirschsprung disease, Long-term functional outcome, Transabdominal- and transanal pull-through

## Abstract

**Background:**

Hirschsprung disease (HSCR) is a common congenital disorder presenting with functional obstruction due to aganglionosis of the colon. There are numerous types of pull-through surgery for managing HSCR, such as transabdominal endorectal (Soave), Swenson, Duhamel, transanal endorectal pull-through (TEPT), and laparoscopic (Georgeson) approach. Here, we aimed to describe the long-term outcome of patients with HSCR who underwent transabdominal Soave, Duhamel, and TEPT in our institution.

**Methods:**

We performed a cross-sectional analysis for patients who underwent Duhamel, Soave, and TEPT at our institution from January 2012 to December 2015. Long-term functional outcome was determined by bowel function score (BFS). The BFS was obtained by interviewing patients who had completed at least three years of follow-up.

**Results:**

Twenty-five patients were included in this study who underwent transabdominal Soave (*n* = 8), Duhamel (*n* = 4), and TEPT (*n* = 13). There were 24 patients with short aganglionosis type. The median age of HSCR diagnosis was 10 (IQR = 1–39) months, while the median age of pull-through surgery was 17 (IQR = 7–47) months. The median follow-up of BFS level for HSCR patients after pull-through was 72 (IQR, 54–99) months. There were 11 patients with good BFS level and 10 patients with normal BFS level. Additionally, 50% of Duhamel patients had poor BFS level, while 50% of Soave patients had good BFS level, and 54% of TEPT patients had normal BFS level (*p* = 0.027). As many as 50% of Duhamel patients showed daily soiling and required protective aids, while 38.5% of TEPT had staining less than 1/week and no change of underwear required, and 50% of Soave patients revealed no soiling, respectively (*p* = 0.030). Furthermore, 75% of Duhamel patients had accidents, while 75% of Soave and 46.2% of TEPT patients had no accidents (*p* = 0.035).

**Conclusion:**

Our study shows that the type of definitive surgery might affect the long-term bowel functional outcome; particularly, the TEPT approach might have some advantages over the transabdominal Soave and Duhamel procedures.

## Background

Hirschsprung Disease (HSCR) is one of the most common congenital anomalies, characterized by failure of neural crest migration in the colon causing aganglionosis starting in the distal part of the colon, resulting in functional obstruction [1]. It is estimated that the incidence is 1:5,000 live birth and more commonly in males by 4 to 1 compared to females [1]. A previous study showed a higher incidence in Indonesia, which is 1:3,250 live birth [2].

A definitive treatment for HSCR is pull-through surgery [3]. Several pull-through surgeries are transabdominal endorectal (Soave), Swenson, Duhamel, transanal endorectal pull-through (TEPT), and laparoscopic (Georgeson) approach. Several studies have reported the functional outcomes of HSCR patients following pull-through [4–8]. However, the studies that compare long-term functional outcomes from these different surgical approaches are very limited [9]. Long-term outcomes are defined by the patient’s ability to control defecation, social function, and quality of life [10]. One of the methods used to measure bowel function as a long term HSCR outcome is the bowel function score (BFS) [11]. Therefore, we aimed to describe the long-term outcomes of patients with HSCR who underwent transabdominal Soave, Duhamel, and TEPT in our institution.

## Methods

### Subjects

This study design was cross-sectional to determine the long-term (three-year or more) functional outcome of HSCR patients. We used the International Statistical Classification of Diseases and Related Health Problems, 10th Revision (ICD-10) code of Q43.1 (HSCR) to determine the patients diagnosed with HSCR. Eighty-six medical records of HSCR patients after pull-through were checked.

Inclusion criteria were HSCR patients who underwent Duhamel, Soave, or TEPT procedures in our institution from January 2012 to December 2015 and had completed at least three years of follow-up. The exclusion criteria were an incomplete medical record, unable to be contacted, refusal to be interviewed, and deceased patients.

The Institutional Review Board approved the study, Faculty of Medicine, Public Health and Nursing, Universitas Gadjah Mada/Dr. Sardjito Hospital (KE/FK/1043/EC/2018). All parents completed written informed consent forms before participation in this study.

### Pull-through surgery

According to previous studies, pull-through surgery was done using either one of the techniques, such as transabdominal Soave, Duhamel, or TEPT procedure [5, 8, 12]. In our center, transabdominal Soave and Duhamel techniques were performed in a two-stage procedure using an initial stoma, while TEPT was a primary pull-through procedure. The type of definitive surgery was chosen according to the preference of the attending pediatric surgeon.

### Bowel function score

Long-term functional outcome was determined using BFS [11]. The score was obtained by interviewing the patient's parents via telephone. The score determines seven variables: scores from 0 to 3, except the frequency of defecation, which is scored 1–2. These variables are the ability to hold back defecations, feels/reports the urge to defecate, frequency of defecation, soiling, fecal accidents, constipation, and social problems. In this scoring system, the fecal incontinence is classified into soiling and accidents. Soiling is defined as staining of the underwear or involuntary loss of small amounts of stool, while accidents are described as involuntary loss of large amounts of stool requiring change of underwear [11, 13]. The maximum score is 20. The result is classified as normal when the score is 18 points or more; good when scored 9–16 points and have occasional staining and infrequent defecation accident; fair when scored 7–11 points with intermittent daily soiling or staining; and poor when scored 6–9 points and had to use daily enemas because of severe constipation or constant soiling [11, 14].

### Statistical analysis

Data were presented as frequency and median (interquartile range, IQR). The association between BFS and type of pull-through was determined using Fisher’s exact test. *p*-value of < 0.05 was defined as significant.

## Results

### Baseline characteristics

**Fig. 1:**
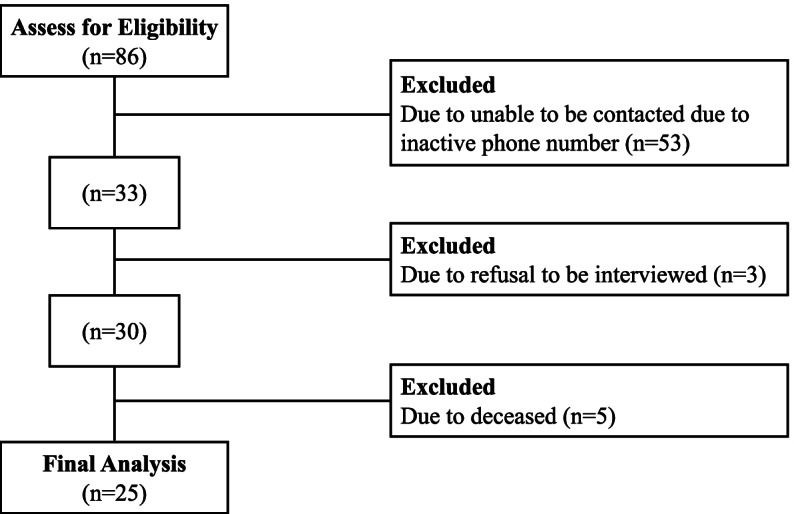
Flowchart of study inclusion and exclusion criteria

Sixty-one patients were excluded due to refusal to be interviewed (*n* = 3), unable to be contacted due to inactive phone numbers (*n* = 53), and deceased patients (*n* = 5). Twenty-five patients were further analyzed in this study (Fig. 1, Table 1). There were 24 patients with short aganglionosis type [15]. The median age of HSCR diagnosis was 10 (IQR, 1–39) months old, and the median age of pull-through surgery was 17 (IQR, 7–47) months old.

**Table 1:** Baseline characteristics of HSCR patients who underwent pull-through in this study

Characteristics	Soave (*n* = 8)	TEPT (*n* = 13)	Duhamel (*n* = 4)
Sex (n, %)			
▪ Male	8 (100)	7 (53.8)	3 (75)
▪ Female	0	6 (46.2)	1 (25)
Aganglionosis type (n, %)			
▪ Short	8 (100)	12 (92.3)	4 (100)
▪ Long	0	1 (7.7)	0
Age at HSCR diagnosis (median, IQR) (months)	9.5 (2.75–26.5)	6 (1–32)	25.5 (12–39)
Age at definitive surgery (median, IQR) (months)	18.5 (13.25–35)	6 (1–37)	43 (29.5–54.75)
Length of follow-up (median, IQR) (months)	80 (59–93)	55 (42–78)	99.5 (88–100)

*HSCR* Hirschsprung disease, *IQR* interquartile range, *TEPT* transanal endorectal pull-through

### Bowel function score

The median follow-up of BFS level for HSCR patients after pull-through was 72 (IQR, 54–99) months. There were 11 patients with good BFS level and 10 patients with normal BFS level (Table 2).

**Table 2:** BFS in HSCR patients after pull-through procedure

BFS	Pull-through			*p*-value	Total
	**Duhamel (n, %)**	**Soave (n, %)**	**TEPT (n, %)**		
Poor	2 (50)	1 (12.5)	0	0.027*	3 (12)
Fair	1 (25)	0	0		1 (4)
Good	1 (25)	4 (50)	6 (46)		11 (44)
Normal	0	3 (37.5)	7 (54)		10 (40)

^*^significant (*p* < 0.05), *BFS* bowel function score, *HSCR* Hirschsprung disease

### BFS in HSCR patients after different pull-through procedures

Next, we determined the BFS in HSCR patients after different pull-through techniques. As many as 50% of Duhamel patients had poor BFS level, while 50% of Soave patients had good BFS level, and 54% of TEPT patients had normal BFS level (*p* = 0.027) (Table 2). Moreover, we analyzed seven components of BFS for each pull-through performed (Table 3). As many as 50% Duhamel patients showed daily soiling and required protective aids, while 38.5% of TEPT patients had staining less than 1/week and no change of underwear required, and 50% of Soave patients revealed no soiling (*p* = 0.030). Furthermore, most Duhamel patients (75%) had accidents, while most Soave (75%) and TEPT (46.2%) patients had no accidents (*p* = 0.035) (Table 3).

**Table 3:** Analysis of BFS components for each pull-through performed

BFS	Pull-through			*p*
	Duhamel (n, %)	Soave (n, %)	TEPT (n, %)	
Ability to hold back defecation				
▪ No voluntary control	1 (25)	3 (37.5)	1 (7.7)	0.383
▪ Weekly problems	1 (25)	0	2 (15.4)	
▪ Problems less than 1/week	1 (25)	0	2 (15.4)	
▪ Always	1 (25)	5 (62.5)	8 (61.5)	
Feels/reports the urge to defecate				
▪ Absent	1 (25)	1 (12.5)	0	0.542
▪ Uncertain	1 (25)	1 (12.5)	1 (7.7)	
▪ Most of the time	1 (25)	1 (12.5)	2 (15.3)	
▪ Always	1 (25)	5 (62.5)	10 (77)	
Frequency of defecation				
▪ More often/less often	2 (50)	3 (37.5)	2 (15.4)	0.310
▪ Every other day to twice a day	2 (50)	5 (62.5)	11 (84.6)	
Soiling				
▪ Daily soiling, requires protective aids	2 (50)	0	0	0.030*
▪ Frequent staining, change of underwear often required	1 (25)	3 (37.5)	1 (23)	
▪ Staining less than 1/week, no change of underwear required	1 (25)	1 (12.5)	5 (38.5)	
▪ Never	0	4 (50)	5 (38.5)	
Accidents				
▪ Daily, require protective aids during day and night	3 (75)	2 (25)	1 (7.7)	0.035*
▪ Weekly accidents; often require protective aids	0	0	1 (23)	
▪ Fewer than 1/week	1 (25)	0	2 (23)	
▪ Never	0	6 (75)	6 (46.2)	
Constipation				
▪ Manageable with enemas	0	0	0	0.181
▪ Manageable with laxative	1 (25)	2 (25)	0	
▪ Manageable with diet	0	1 (12.5)	0	
▪ No constipation	3 (75)	5 (62.5)	13 (100)	
Social problems				
▪ Several social and/or psychic problem	0	0	0	0.647
▪ Problems causing restrictions in social life	0	0	0	
▪ Sometimes (foul odors)	1 (25)	1 (12.5)	1 (7.7)	
▪ No social problems	3 (75)	7 (78.5)	12 (92.3)	

^*^significant (*p* < 0.05), *BFS* bowel function score, *HSCR* Hirschsprung disease

## Discussion

Here, we show that most of our patients have good long-term functional bowel outcomes following transabdominal and transanal pull-through surgeries. Previously, we determined the functional outcomes after several definitive surgeries [5, 8]. However, there are some novelties in this study: 1) we determined the long-term outcomes in three different techniques (*vs.* two procedures [5] *vs.* one method [8]); 2) we included the transabdominal and transanal approaches (*vs.* two transabdominal procedures [5]), and 3) at least three years' follow-up after pull-through (*vs.* no defined time of follow-up [5, 8]). Our study provided another report of the functional bowel outcomes between three different pull-through techniques from a different population, *i.e.*, Indonesia (*vs.* a Western country [9]). Another strength of our study is a prospective design and the length of follow-up of at least three years after definitive surgery. Several studies reported the long-term functional outcomes, mostly from Western countries [16–20]. Our study provided an additional critical report on the long-term bowel function from a South-East Asian country, i.e., Indonesia. A recent study showed that although impairment of functional outcomes is common after surgery, intestinal function was improved following the patient's age [17]. In contrast, other studies reported that the impairment of bowel function does not change with increasing age [7, 18]. They suggested that continuous follow-up and management is essential for these patients’ groups [18].

In our study, patients who underwent transabdominal Duhamel and Soave procedures might have a poorer functional bowel outcome than patients who underwent TEPT (Table 2). There are several advantages of TEPT over transabdominal procedures, including its minimally invasive approach, better cosmetic result, and avoidance of abdominal contamination [21]. Moreover, there is a recognized heterogeneity of operative time and hospital stay, implying variation of skills of the surgeons and postoperative care in associated hospitals [22]. Long-term functional outcomes of HSCR patients who underwent pull-through surgery vary and are deemed due to the operator's experiences. Thus, preferences on the choices of the pull-through technique rely significantly on the operator [23].

Interestingly, our study also reveals that type of definitive procedures might influence the incidence of soiling and accidents (Table 3). There is still conflicting evidence on which procedure is associated with soiling. Soiling in postoperative HSCR patients is due to damaged sensation and sphincter mechanism in primary repair or affected colonic motility after resection of rectosigmoid, the fecal reservoir. These can be seen with damaged/absence of anal canal and/or sphincters due to improper surgical technique in both approaches, either transabdominal or transanal [24]. Fecal incontinence risk could be reduced by creating anastomoses higher than the dentate line [25]. This fecal incontinence could improve over time, but some patients with more severe symptoms require redoing the pull-through [26].

In this study, we used the BFS, not the Krickenbeck classification, to determine the long-term outcomes of patients with HSCR after pull-through. While the Krickenbeck classification is easily applied in clinical practice [2, 5, 8], it is originally designed for patients with anorectal malformation (ARM). It should be noted that there are some differences between HSCR and ARM. Patients with HSCR show normal anal canal and sphincter and usually do not reveal the spinal cord and vertebrae anomalies [5].

Some weaknesses of our study are noted, such as 1) small sample size; 2) an unequal number of patients between three procedures; and 3) we only determined the functional bowel outcomes and the type of pull-through surgery according to overall means without considering other factors that might affect the findings, including sex, degree of aganglionosis, surgeons’ skills, age at HSCR diagnosis, age at definitive surgery, and coexisting dysganglionoses. In addition, a recent study showed that age at definitive surgery, *i.e.*, neonatal vs. delayed primary pull-through, did not affect the functional bowel outcomes [16].

## Conclusions

Our study shows that the type of definitive surgery might affect the long-term bowel functional outcome; particularly, the TEPT approach might have some advantages over the transabdominal Soave and Duhamel procedures.

## Data Availability

All data generated or analyzed during this study are included in the submission. The raw data are available from the corresponding author on reasonable request.

## Abbreviations

BFS: Bowel function score
HSCR: Hirschsprung disease
TEPT: Transanal endorectal pull-through
